# Comparative study on skill and health related physical fitness characteristics between national basketball and football players in Sri Lanka

**DOI:** 10.1186/s13104-019-4434-6

**Published:** 2019-07-12

**Authors:** Anula Kariyawasam, Anoja Ariyasinghe, Arun Rajaratnam, Padmakanthi Subasinghe

**Affiliations:** 1 Department of Physiology, Faculty of Medicine, University of Peradeniya, Peradeniya, Sri Lanka; 2 National Hospital of Sri Lanka, Colombo, Sri Lanka; 3 National Institute of Sports Medicine, Colombo, Sri Lanka

**Keywords:** Fitness profile, Endurance, Strength, Flexibility

## Abstract

**Objective:**

To compare health and skill related physical fitness profiles between healthy, male, basketball and football players of Sri Lankan national teams.

**Results:**

Thirty basketball players (mean age 24 ± 4.5 years) and 30 football players (mean age 23 ± 4.3 years) were evaluated for health related fitness characteristics (body fat percentage, cardio-respiratory fitness, isometric hand grip strength, lower body and upper body muscular strength, abdominal and upper body muscular endurance, and flexibility) and skill related fitness characteristics (agility, speed, explosive throwing power, jumping power, reaction time, coordination, static balance). Fat percentage, upper body endurance, grip strength, running speed, explosive power, jumping power, balance and coordination were significantly higher in basketball players than in footballers. Football players had better upper body strength, flexibility, reaction time and agility than those of basketball players. The latter two were statistically significant. Basketball players had better mean lower body strength, although not significant. Fitness characteristics were different between basketball and football players. The results have implications in tailoring training activities to improve relevant fitness characteristics.

## Introduction

Success of team sports require psychological and physical well being in addition to precise motor skills, tactical qualities, playing style, seasonal period, individual and team motivation [1]. Of the determinants affecting sports performance, physical fitness may be the most important [2]. Physical fitness is defined as the capacity to perform daily activity with vitality and sharpness, without undue fatigue while being able to appreciate recreation time interests and to meet the unpredicted emergencies [3]. It is the combination of health and skill related aspects of physical fitness which is imperative in shaping individuals in sports or games. Basketball and football are two competitive sports, both of which demand high degree of physical fitness from their players for easy and efficient execution of technical and tactical skills mastered by the players.

Fitness components related to health are body composition, cardio-respiratory fitness, flexibility, muscle strength and muscular endurance. The motor potential to carry out physical activity with regard to speed, agility, power, balance, coordination and reaction time is described by skill related physical fitness [4].

Sri Lankan basketball and football teams receive relatively less recognition and achievements in the international arena, and this is probably due to deficiencies in player training programmes. With only a limited number of studies comparing fitness profiles of both sports [5–8] and only one from Sri Lanka [9] we intended to assess and compare health and skill related physical fitness characteristics of players of Sri Lankan national level basketball and football teams.

Our study is unique that it has evaluated all components of physical fitness including flexibility, balance, coordination and reaction time, which have not been studied extensively. Also, it is one of the few studies performed on national teams. Improving the quality of the players to internationally recognized standards with the limited resources available is a current necessity in Sri Lanka. Our study aimed to assess and compare health and skill related physical fitness profiles between healthy, male, basketball and football players of Sri Lankan national teams.

## Main text

### Materials and methods

#### Sample population and study design

A cross-sectional study using a sample of 60 healthy national level, basketball (n = 30) players and football (n = 30) players who were at pre-competition stage and participated in the assessment of physical fitness conducted by the institute of sports medicine, ministry of sports in Sri Lanka were recruited. Players in each team had more than 6 years of playing experience and received the same type of training for 3 days a week (4 h a day), nutrition and supplements for a similar duration. The participants were advised to refrain from strenuous exercise for at least 48 h and not to consume a heavy diet prior to fitness testing. Each fitness test was performed after warming up sessions of jogging and stretching as recommended. Necessary precautions were taken for injury prevention during tests.

### Data collection

#### Health related physical fitness characteristics

Body fat percentage was calculated by measuring skin fold thicknesses in the chest, abdomen and thigh [10] using the Harpenden skinfold caliper (CE 0120, China) and recorded to the nearest mm. Cardiorespiratory fitness was assessed with multi-stage shuttle run test/Beep test (intermittent) asking the player to run in-between two lines 20 m apart. Player had to increase the velocity of running until the maximum possible speed achieved. The VO_2_max (maximum oxygen consumption; ml/min/kg), an indicator of cardiorespiratory fitness was calculated using the speed at maximal successful level [11].

Hand grip strength was measured using adjustable digital handgrip dynamometer (EH 101, ISO 9001, China) following standard technique. Best of three grips for each hands obtained in kg. Upper body and lower body muscular strength were measured by one repetition maximum test (1RM) with 1-RM bench press (SH 5063, Olympic) and 1-RM leg press machine (SH 5070), respectively. The player after appropriate warm up had to lift 40% to 60% of maximum lifting weight in repetition with rests in between lifts. Maximum weight in kg lifted or pressed was recorded [13]. Abdominal endurance and upper body endurance were assessed by 1 min sit-ups and 1-min push-ups, respectively [13]. Number of properly completed sit ups/pushups in a minute was recorded. Flexibility was assessed with sit and reach test. With both bare feet against the sit and reach box, the participant was asked to bend forward with both hands keeping on top of each other. Average measurement of three attempts was recorded.

#### Skill related physical fitness characteristics

Sprint speed was assessed with 40 m sprint test using time gate system, as suggested [12]. The time taken was recorded to the nearest 0.01 s. Agility was tested using 5-10-5 yard pro agility test using the protocol outlined by Gökdemir et al. [5]. The player was asked to sprint 5 yards to the right from the starting line, touch the line, sprint 10 yards to the left, touch the line and turn back to the right and sprint 5 yard to the start line. The time taken to sprint between these two points was recorded. The vertical jump test was performed asking the player to stand on a jumping platform and reach up against the wall as high as possible. This height was standing reach height. Then, the player was instructed to perform a vertical jump with their maximal effort. Maximal reaching distance was recorded. The difference between the standing reach height and maximal reaching height during jump were calculated.

Medicine ball throw was used to assess explosive power in a horizontal direction [13]. The subject in a seated position held the medicine ball with both hands and forcefully threw it as far as possible keeping the head, shoulders and hips supported against a wall. Average distance in three attempts was taken. Time which the participant could maintain stork balance posture was used to test the static balance [13]. Coordination was tested with wall catch test. Number of catches completed in a minute was recorded. The reaction time was assessed by the drop stick test [14].

#### Statistical analysis

Statistical analysis was performed using SPSS for Windows, version 20.0. Data were presented as mean ± standard deviation (SD). Age adjusted analysis was done in both teams. Two tailed independent sample *t* test was used for analyzing differences of means. A p value ≤ 0.05 was taken statistically significant.

### Results

The mean age of basketball players was 24 years ± 4.5 years (range 18–34 years) and of football players was 23 ± 4.3 years (range 17–36 years). The mean heights of basketball and football players were 183.33 ± 8.39 cm and 172.83 ± 6.23 cm, respectively. The mean weights of basketball and football players were 79.33 ± 12.87 kg and 63.67 ± 8.31 kg, respectively.

As shown in Table 1, mean fat percentage, upper body endurance and grip strength were significantly higher in basketball players compared to footballers (p < 0.01). Basketball players showed higher abdominal endurance and lower body muscle strength not significant statistically (p > 0.05). Although not significant upper body strength and flexibility were higher in footballers compared to basketball players (p > 0.05). However, the difference in VO_2_ max between the two groups was negligible.

**Table 1:** Comparison of health related fitness parameters between basketball and football players

Heath related fitness variable	Basketball players (Mean ± SD)	Football players (Mean ± SD)	p value
Fat percentage	11.61 ± 5.48	8.59 ± 4.15	0.019*
Cardiorespiratory fitness [VO_2_ max (ml/kg/min)]	50.6 ± 4.0	52.0 ± 4.0	0.181
Muscular endurance (Sit-ups per 1 min)	49.63 ± 6.77	46.40 ± 7.69	0.089
Muscular endurance (Push-ups per 1 min)	47.03 ± 14.78	37.77 ± 8.08	0.004*
Grip strength—left hand (kg)	42.59 ± 7.39	38.03 ± 5.92	0.011*
Grip strength—right hand (kg)	46.08 ± 6.83	39.54 ± 6.49	< 0.001*
Upper body muscle strength (kg) [1-RM bench press]	111.60 ± 64.09	119.10 ± 19.13	0.542
Lower body muscle strength (kg) [1-RM leg press]	219.46 ± 75.07	196.76 ± 30.03	0.13
Flexibility (cm) [Sit and reach test]	10.87 ± 5.31	12.73 ± 5.46	0.185

*SD* standard deviation

* Statistically significant

As shown in Table 2, basketball players had significantly higher mean agility, muscle power coordination, balance compared with football players (p < 0.001). Speed was significantly lower in basketball players than the football players (p = 0.01). Footballers were significantly better at reaction time compared to the basketball players (p < 0.05).

**Table 2:** Comparison of skill related physical fitness parameters between basketball and football players

Skill related fitness variable	Basketball players (Mean ± SD)	Football players (Mean ± SD)	p value
Sprint speed (s) (40-m sprint test)	5.48 ± 0.50	5.91 ± 0.41	0.001*
Agility (s)—pro agility test	5.98 ± 0.33	5.39 ± 0.49	< 0.001*
Medicine ball throw (m)	7.61 ± 1.08	6.09 ± 0.53	< 0.001*
Vertical jump—both legs (cm)	47.97 ± 6.82	37.9 ± 6.22	< 0.001*
Vertical jump—left leg (cm)	34.87 ± 6.40	32.57 ± 8.63	0.24
Vertical jump—right leg (cm)	37.80 ± 8.02	28.57 ± 6.60	< 0.001*
Coordination—wall catch per minute	51.30 ± 6.08	40.37 ± 8.99	< 0.001*
Static balance (s)	51.97 ± 12.96	15.07 ± 1.74	< 0.001*
Reaction time (s) drop stick test	0.12 ± 0.02	0.11 ± 0.01	0.014*

*SD* standard deviation

* Statistically significant

### Discussion

The mean ages of our study group is comparable to similar studies in Turkey [6]. The fat percentage of our football players was in par with what is expected in professional international footballers [15]. Compared to the basketball players, our football players had significantly lower fat percentage supporting others [6] and this may be attributed to the predominant aerobic nature of their sport, but also may reflect less fat intake.

Cardiorespiratory fitness (VO_2_max) is crucial in football.. In the present study, VO_2_max in footballers was not significantly different from that of basketballers and our players had a lower VO_2_max compared to the international players [16]. Elite football players are known to have a VO_2_max above 60 ml/kg/min [15]. In the present study, mean VO_2_max of basketball players was lower than 55 ml/kg/min and even lower than 55 ml/kg/min of VO_2_ max may be considered as adequate for elite basketball players [17, 18]. Isometric grip strength of both hands was higher among basketball players and may be important as various movements during catching, holding, shooting and throwing the ball, rely on the wrist and digital flexor activity. Upper body muscular strength of football players was similar to others [19]. Upper body strength and endurance of our football players were lower than those of elite players [20]. Contrary to what we expected, lower body strength required for kicking, running and jumping in our footballers was insignificantly lesser than that of basketball players. Abdominal endurance was better among basketball players.

Flexibility is an important component in both games, and may help in achieving greater speed and agility in football and better jump heights and defenses in basketball. Our football players were more flexible than basketball players, supporting the findings of Theoharopoulos et al. [21].

Basketball players exhibited relatively better speed, explosive power, balance and coordination. Basketball is one of the fastest game, thus players require excellent speed for better performance [3]. Srinet [7] showed that sprinting speed of Indian college basketball players significantly better than football players. Our national level football players had better speed at 40 m sprint test compared to Indian college footballers [22]. Our football players had better agility and is fundamental to good performance in football.

As expected, basketball players showed better explosive power than footballers, but much lower compared to most international studies [12]. A higher level of muscular power in basketball players reduces the injury risk [23]. The lower jumping power in footballers may be due to relative shortness or due to inadequate lower limb training. Medicine ball throwing power was higher among basketball players, reflecting the necessity of throwing skill in basketball players compared to footballers.

Both static balance and coordination were better among basketball players. This contradicts the works of others [5, 7]. Good balance will help players to keep their shots on target, reach and play difficult strikes with accuracy and also to maintain posture when a defender tries to gain possession of the ball [24]. Reaction time is often a neglected component in both sports. Similar to ours, Atan and Akyol [8] also showed that reaction time to be better in footballers than in basketball players. Previous studies have shown goal keepers to have better reaction time compared to other football positions [25].

Our players may not be elite in par with international standards, but have fitness components higher than those reported for general Sri Lankan population [26]). The players in Sri Lankan national basketball and football teams were deficient in certain fitness components compared to international elite counterparts. This emphasizes the necessity of having relevant scientifically based training regimes in preparing these players for international events. It should be noted that the level of strength and endurance training influences [27], and can contribute to the differences in physical fitness parameters observed between the two teams of players.

### Conclusions

Sri Lankan national level basketball players had better upper body endurance, abdominal endurance isometric grip strength, lower body strength, running speed, explosive throwing power, vertical jumping power, balance and coordination. The national level football players had better VO_2_max, upper body strength, flexibility, reaction time, agility and lower fat percentage. The results have implications in tailoring appropriate training activities to improve relevant fitness characteristics.

## Limitations

To the authors’ knowledge, this is the first study attempting to assess physical fitness characteristics and compare them between basketball and football players in Sri Lankan population. The present study is limited to a small sample of players. These findings cannot be generalized. Physical fitness parameters will be different in players of different positions. However, it was not considered in the present study as the sample is small.

## Data Availability

The data sets used and/or analyzed during the current study are available from the corresponding author without limitation.

## Abbreviations

1RM: one repetition maximum
VO_2_max: maximum oxygen consumption
